# Supplementary material for: Are carbon emissions trading and green financial instruments synergistic? -Comprehensive quantitative research based on content analysis
Source: PLoS One. 2024 Mar 7;19(3):e0298601. doi: 10.1371/journal.pone.0298601 (PMC10919601; doi:10.1371/journal.pone.0298601)
Supplement: S1 Data — (ZIP) [file pone.0298601.s001.zip › Data/地级市Z_F工作报告.zip!/╒π╜¡/╓█╔╜╩╨/└┤╫╘╣1⁄2╓┌║┼í╛┬φ┐╦╩2╛▌═°í┐_2016.docx]

各位代表：

现在，我代表市人民政府向大会报告工作，请予审议，并请政协委员和其他列席会议的同志提出意见。

一、“十二五”发展情况和2015年工作

过去五年是极不寻常的五年。在省委、省政府和市委的正确领导下，在市人大、市政协的监督支持下，我们紧紧团结依靠全市人民，贯彻落实党的十八大和十八届三中、四中、五中全会精神，深入贯彻习近平总书记系列重要讲话，认真执行省委、省政府和市委各项决策部署，凝心聚力、克难攻坚，新区建设取得明显成效。

舟山开发建设上升为国家战略。2011年6月30日，国务院批准设立浙江舟山群岛新区。此后相继批复新区发展规划、舟山港综合保税区，建立部省际联席会议制度。习近平总书记亲临舟山视察并作出重要指示。李克强总理亲自点题建设舟山江海联运服务中心和绿色石化基地。国家部委通过各种方式支持新区建设，首期新增12万亩建设用地规划指标。省委、省政府举全省之力推进新区开发建设，出台推进新区建设的若干意见，批复新区建设三年行动计划，赋予省级经济管理权限，7.4万亩基本农田省内统筹政策正式落地。

综合实力再上台阶。全市生产总值从645亿元增加到1095亿元，年均增长9.9%，增速位居全省首位。人均生产总值从5.84万元增加到9.53万元。一般公共预算收入从61亿元增加到112.7亿元，年均增长13.1%。海洋经济增加值从431亿元增加到766亿元，年均增长11.8%。三次产业比例从9.8：45.3：44.9调整为10.2：41.4：48.4，经济综合实力持续增强。

新区建设拉开框架。组织实施省市重点项目299个，长宏国际产业园、浙能六横电厂、多端柔性直流输电示范工程、北向疏港公路、大陆引水二期等重大项目建成投用。新区（城市）总体规划获省政府批复实施，市、县（区）、乡镇（街道）三级土地利用总体规划全面获批。城市建成区面积达到71.9平方公里，城镇化率达到66.9%，提高3.3个百分点。经济功能区初步发挥了生力军作用。累计完成固定资产投资3947亿元，年均增长23.3%；引进市外资金1095亿元，实际利用外资7.8亿美元，引进世界500强企业12家。

先行先试取得突破。实施以“三强三优”为主要内容的新区行政体制创新，市政府工作部门、市级事业单位分别精简26.5%和24.8%。“四张清单一张网”建设取得良好成效，市场监管、海洋综合行政执法和旅游综合改革等走在全国前列。城乡发展一体化改革有效推进，渔农村综合配套改革政策稳步实施。深化资源要素配套改革，设立船舶等产业投资基金，成立海域海岛使用权储备交易中心、海洋资源收储公司。扩大对外开放，舟山港综合保税区本岛分区封关运作，口岸开放面积达到1344.8平方公里。加强科技人才工作，海洋科学城等创新平台逐步形成，累计实施各级各类科技项目2726项。引进科创领军人才（团队）企业92家、各类紧缺人才7410名、国家“千人计划”专家4名、省“千人计划”专家18名。百人计划成效明显。区域合作不断深化，援藏、援疆等对口支援工作成绩显著。

城乡发展步伐加快。城市建设不断推进，主城区市政配套和综合商务功能进一步提升。旧城有机更新步伐加快，县城、小城市和中心镇建设扎实推进。建成89个渔农村特色精品社区，在全省率先实现美丽乡村创建全域化。强力推进“三改一拆”，完成“三改”面积787万平方米、拆违面积552万平方米。深入实施“五水共治”，治理黑臭河70条118公里，全面完成渔农村生活污水治理，21.7万渔农民饮水条件得到提升改善。加强生态环境保护，空气质量全国领先。强势推进污染行业整治，基本淘汰黄标车，节能减排降耗顺利完成省下达任务。舟山渔场修复振兴暨“一打三整治”行动扎实推进，取缔涉渔“三无”船舶2123艘，增殖放流各类苗种20亿尾（粒）。成功创建国家环保模范城市、国家节水型城市。城乡统筹发展水平居全省第三位。

民生事业持续改善。财政用于民生支出602亿元，年均增长16.9%。城乡居民收入分别达到44845元和25903元，收入比从1.84:1缩小到1.73：1。新增市级菜篮子蔬菜基地7450亩，总面积达到1.8万亩。改造渔农村困难群众危旧房2031户，建成保障性住房26608套。就业社保成效明显，新增城镇就业人员5.7万人，14.7万被征地农民转轨参加职工养老保险，2.1万原集体捕捞渔民享受生活补贴。海岛特色养老服务体系建设富有成效，城乡居民基础养老金从每月70元提高到145元。

社会建设不断进步。实现义务教育基本均衡化创建目标，普惠性幼儿园覆盖面达到78%，校安工程全面完成，职业教育完成布局、实现提升。实施公立医院综合改革，基层医疗卫生服务实现更高水平覆盖。办好中国海洋文化节，在全国首创“淘文化”平台，建成渔农村文化礼堂124家。巩固国家卫生城市创建成效，荣获全国文明城市提名资格和全国双拥模范城六连冠。广电、新闻出版、档案、史志、文物和非遗保护等事业全面发展。发挥工青妇等群团组织作用，民族、宗教、外事、侨务、老龄、儿童、红十字、慈善和残疾人事业等取得新成绩。食品药品质量安全总体平稳向好。安全生产三项指标保持零增长。深化“网格化管理、组团式服务”，加强普法、信访等工作，严厉打击违法犯罪，群众安全感满意率居全省前列,连续十年被省委、省政府授予“平安市”称号，荣获平安银鼎。

自身建设得到加强。深入开展党的群众路线教育实践活动和“三严三实”专题教育。大力推进法治政府和服务型政府建设，自觉接受人大、政协监督，共办复市人大代表议案建议779件、政协提案1802件。建立府院联席会议制度，重视发挥决策咨询委和新区发展顾问的作用。开展电视问政、网络问政，推进政务公开和政府信息公开。加快转变政府职能，促进廉洁政府建设，组建公共项目建设中心、造价中心、审计中心。坚决执行中央“八项规定”精神，“三公”经费支出逐年压缩。加大反腐倡廉力度，重视行政效能建设，累计查处各类违规违纪、不作为等行为人员1142人。

2015年，面对复杂严峻的经济形势，我们牢牢把握新区发展的阶段性特点，迎难而上，精准发力，扎实推进各项工作，较好地完成了市六届人大五次会议确定的目标任务。地区生产总值增长9.2%；一般公共预算收入增长11.6%；完成固定资产投资1135亿元，增长18.1%；规上工业总产值达到1681.9亿元，增长10%；港口货物吞吐量3.79亿吨，增长9.3%；旅游总收入552亿元，增长15.7%；实现渔农业总产值222亿元，增长5.6%。

加快建设一中心四基地。舟山江海联运服务中心建设边谋边干，总体方案近期有望获国务院批复；江海联运产业投资基金全面运作，浙江石油化工交易中心挂牌成立，公共信息平台上线运行，建立中国船级社舟山研发中心并启动新型江海联运船型设计和标准制定。绿色石化基地项目实质性推进，浙江石油化工有限公司注册落地。国际海事服务基地建设初见成效，成立外轮供应服务中心，保税燃料油供应跃居全国港区第二位。中国（浙江）大宗商品交易中心获国务院批复，现货交易额超过400亿元。国际海岛旅游大会影响广泛，舟山成为永久性会址。国家远洋渔业基地获农业部批准，远洋渔业产量达到46.5万吨。

扎实推进稳增长调结构。抓好国家船舶与海工装备新型工业化示范基地建设，8家重点船企的订单和完工量占全市九成以上，规上船舶工业产值达到860亿元，造船三大指标占全国比重均超过10%。推进北斗海洋通信产业园等建设，海洋电子产业产值超过30亿元。规上高新技术产业增加值达到159.3亿元，增长8%。建筑和房地产业稳定发展，建筑业产值达到226.5亿元，增长7.8%。观音文化园正式开工，白沙等精品旅游示范岛建设扎实推进，国际邮轮港和水上飞机投入运营，普陀山朱家尖景区加快提升。获批赴台个人游试点城市。加强企业帮扶解困，为企业减负41亿元，龙头骨干企业保持平稳，资金链、担保链风险基本可控。

坚定不移强改革增活力。贯彻实施省海港整合与管理体制调整决策，宁波舟山港一体化实质性推进，省海港集团落户舟山。实施项目中心制，开展县（区）、功能区“比学赶超”活动。推进普陀山朱家尖一体化管理，实施海洋产业集聚区（核心区）共建共享办法。整合市属经营性国有资产，重组商贸集团和国有航运企业，打造四大国有投资公司。成功发行全国首单非上市企业的公司债。实施城镇低效用地再开发4652亩，盘活存量建设用地2748亩。渔农村集体资产股份合作制改革和户籍制度改革试点基本完成。实行排污权有偿使用和主要污染物总量控制激励制度。

全力以赴促统筹惠民生。十方面为民实事项目全面完成。浙江大学舟山校区、舟山技师学院建成投用，浙江海洋学院更名浙江海洋大学进入公示阶段，新区旅游与健康职业学院正式建校。瑞金医院舟山分院项目进展顺利，舟山群岛网络医院上线启用。在全省率先实现低保标准城乡并轨。举办舟山群岛国际马拉松等精品赛事。加快危房改造，城区D级危房全部列入改造计划。深入开展水路货运船舶、油品储运设施等专项整治。建立新区社会公共服务与监管中心。取得抗击强台风“灿鸿”正面袭击的重大胜利。

同时，我们清醒地看到存在的问题和不足：在宏观经济环境持续低迷的背景下，超常规推进新区建设力度不够，地区生产总值、规上工业总产值、外贸等指标未能实现“十二五”规划目标；重大项目落地不多、推进不快，经济功能区的作用有待进一步发挥，支撑新区持续发展的动力还不够强劲；改革开放仍需进一步先行突破；民生改善还有许多不尽人意的地方，人民群众的获得感还不明显；各级政府及其工作人员的理念、效能、作风，还不能很好地适应新区快速发展要求。对此，我们要高度重视，采取切实措施努力解决。

各位代表，过去五年为“十三五”发展打下了重要基础。在此，我代表市人民政府，向在各行各业辛勤劳动的全市人民，致以崇高的敬意！向给予政府工作支持和监督的人大代表、政协委员，表示衷心的感谢！向驻舟人民解放军、武警部队官兵，向在舟部省属单位，向各民主党派、工商联、人民团体和社会各界人士，向所有投资者、建设者，向关心和支持我市现代化建设的港澳台同胞和海内外朋友们，表示衷心的感谢！

二、“十三五”发展目标和创新突破的重点

根据市委“十三五”规划建议，市政府制定了《舟山市国民经济和社会发展第十三个五年规划纲要（草案）》，提请大会审议。经本次大会批准后，我们将认真组织实施。

“十三五”是充满机遇、充满挑战的五年。国家实施“一带一路”、长江经济带战略和建设海洋强国，舟山在保障国家经济安全、先行探索海洋开发保护中的地位更加突出；国家部委以及省委省政府持续加力支持新区建设，舟山大开发大建设的条件正在形成，新区进入了负重奋进、创新突破的关键时期。

“十三五”发展的指导思想是：高举中国特色社会主义伟大旗帜，坚持以马克思列宁主义、毛泽东思想、邓小平理论、“三个代表”重要思想、科学发展观为指导，深入贯彻习近平总书记系列重要讲话精神，以“四个全面”战略布局为统领，以创新、协调、绿色、开放、共享发展理念为引领，以“八八战略”为总纲，以海洋经济为主攻方向，坚持发展第一要务，一以贯之落实新区发展规划，以创新驱动、开放带动、改革推动为核心，以增强人民群众获得感为落脚点，全面推进经济建设、政治建设、文化建设、社会建设、生态文明建设，高水平全面建成小康社会，实现新区跨越式发展。

“十三五”发展的总体目标是：到2020年，全市生产总值年均增长10%以上，人均生产总值超过14万元；一般公共预算收入达到165亿元，年均增长8%；完成固定资产投资超过8500亿元；海洋经济增加值占生产总值的比重达到72%；研发经费支出占生产总值比重提高到1.8%以上；新区经济实力实现新跨越，“四岛一城一中心”展现新气象，人民生活迈上新台阶，社会治理达到新水准，建设魅力新舟山。

推进“十三五”发展，必须坚持先行先试、产业支撑、生态优先、千岛共荣、军民融合；必须把负重奋进、创新突破作为政府工作的主基调；必须追求高水平、推进均衡化、增创新优势，集中力量抓好既该干又能干成的大事。

建设舟山江海联运服务中心。发挥舟山通江达海的优势，构筑以“水水中转”为特色的大宗商品及集装箱中转运输体系，为长江经济带提供便捷、高效和低成本的物流服务；培育提升大宗商品交易功能，打造大宗商品储备加工交易基地，增强国家大宗商品战略储备能力；推进宁波舟山港实质性一体化，促进资源整合和港产联动，建设我国港口一体化改革发展示范区，力争舟山港域货物吞吐量达到6亿吨，加快打造国际物流枢纽岛。

构建全方位立体式开放格局。规划建设以油品储备、加工及投资贸易自由化为特色的自由贸易试验区，探索实施既符合国际惯例、又具有区域特色的自由贸易制度，打造我国新一轮对外开放的海上门户。加快开放型经济转型升级，提高舟山港综合保税区建设水平，推进监管模式改革创新，促进区内区外联动发展。创新区域合作模式，加强与上海等长三角及长江沿岸城市的深入合作，积极融入宁波都市区，共享同城效应，实现协同发展。

培育千亿百亿海洋产业集群。坚持集群发展，以引领性、创新性、突破性项目为龙头，构筑现代海洋产业体系。大力培育港贸物流、绿色石化、船舶与海洋工程装备、海洋旅游四大千亿产业集群，加快延伸产业链，提升价值链，增强海洋经济核心竞争力。着力培育现代航空、海洋电子信息、水产品精深加工和远洋渔业等若干百亿产业，提升新区海洋经济的厚度和韧性。加快培育海洋生物医药、海洋新能源、健康养生、文化创意等新兴产业，创新发展现代海洋服务业。

打造海上花园城。优化“一体一圈五岛群”规划布局，统筹推进岛屿开发与保护，增强主要岛屿和中心镇的承载力。按照本岛“南生活、中生态、北生产”的空间格局，推动城市建设向新城集聚，规划建设小干岛和南部滨海大道，加快形成高能级、紧凑型、现代化的中心城区。常住人口城镇化率达到70%以上。加强生态环境整治、修复与保护，建设天蓝、地绿、水净、气爽的美丽家园，打造美丽中国的舟山样本。坚持陆海统筹，以交通为重点，加快构建功能齐全、安全高效的现代基础设施体系。加快甬舟铁路、六横公路大桥等建设，深化沪舟大通道、本岛轻轨等前期研究，完善岛际便捷交通，构筑本岛立体交通体系。推进普陀山机场扩能改造和对外开放。统筹推进电力、水利、信息、围垦、气象和防灾减灾设施建设，提升新区要素综合保障能力。

全面深化改革和实施创新驱动战略。促进军民融合深度发展，推动经济建设与国防建设相协调，争创国家军民融合创新示范区。完善新区行政管理体制和功能区动力机制。围绕供给侧结构性改革，创新行政审批制度和资源要素、金融等体制，充分激发市场主体活力。突出创新驱动，加快推进海洋科技创新，加大海洋科学城等平台建设力度，争创海洋特色鲜明的国家级高新区。深化与大院名校的合作，发挥在舟高校的创新生力军作用。促进产学研协同创新，完善重点产业、重点领域科技攻关联盟机制，推进成果转化应用。深入实施人才优化发展战略，优化人才环境，构筑海洋创新创业人才高地，营造浓厚的创新创业氛围。

增强人民群众获得感。促进城乡居民普遍持续增收，新增城镇就业5万人，城乡居民收入与经济增长基本同步。提高公共服务的普惠性，促进教育、卫生医疗、文化体育等基本公共服务均等化，努力提供广覆盖、保基本、多层次的公共产品，社会保险由制度全覆盖向人员全覆盖转变。通过努力，让人民群众享有更优质的教育、更稳定的工作、更满意的收入、更可靠的社会保障、更高水平的医疗服务、更完善的养老体系、更丰富的文化生活，让新区改革发展成果更多、更公平、更实在地惠及全市人民。

三、2016年的工作任务

今年是“十三五”的开局之年，必须确保经济社会平稳较快发展。综合考虑各方面的因素，建议全市经济和社会发展的主要预期目标为：地区生产总值增长12%以上，固定资产投资1305亿元，增长15%，一般公共预算收入增长10%，城乡居民人均可支配收入分别增长10%和10.5%，节能减排降碳指标完成省定任务。

完成上述目标任务，要按照“项目中心制、工作项目化、任务清单式”的运作模式，战略上打持久战，战术上打歼灭战，聚精会神抓落实，跑出新区加速度。

（一）全力抓好三件战略性大事。找准新区开发建设与服务国家战略的契合点，加快新区突破发展。

加快舟山江海联运服务中心建设。加快海进江、江出海公共泊位建设，开工建设鼠浪湖矿砂二阶段、小洋山北侧内支线码头等项目。争取江海联运特定航线目标船型研发取得突破。促进航运业稳定健康发展，培育壮大龙头航运企业，发展国际一程运输和江海联运，推进货物运输本地化。加快江海联运公共信息平台建设，实现港口物流信息全面对接、有效共享。提升国际海事服务，全面突破保税油不同税号下调和，实现跨关区、跨港区直供，推动“一船多能”外轮供应业务常态化，建设外籍船舶修理服务基地。

扎实推进绿色石化基地建设。坚持“国际一流、产业集群、绿色环保、混合经济”，认真做好规划环评等支撑性专题，争取基地总体规划尽快获批。加快主体项目报批进度，尽早开工建设。加快围垦造地工程，完善交通、水电等基础设施配套，启动鱼山大桥建设。规划石化配套产业园区。

规划建设自由贸易试验区。加强与国家有关部委的沟通对接，全力争取舟山进入全国第三批自由贸易试验区行列。深化自由贸易试验区实施路径的细化研究，做好相关人才的招引储备工作。以舟山港综合保税区为载体，开展自由贸易政策的探索实践。

（二）实施项目攻坚行动。强化“项目中心制”，抓好17个“三重”项目和155个省市重点项目，切实扩大有效投资。

加快重大项目建设。开工建设500千伏联网输变电、神华国华2×66万千瓦机组、大陆引水三期等项目。加快推进富翅门大桥、329国道改造提升、秀山大桥、观音文化园、岱山北部围垦、沈家湾旅游集散中心等建设。完成国家石油储备基地扩建、定马公路改建、蛇移门航道工程等项目。服务推进外钓岛光汇油库、国电海上风电、黄泽山石油储运中转、洋山国际中转物流园区一期、恒安泰海洋柔性管道等重大产业项目建设。

全力推进招商落地。强化精准招商、专业招商、产业链招商，提高招商引资的有效性和针对性。统筹做好对接央企、引进外资、激发民资、浙商回归等工作。全力争取波音飞机装备及交付中心项目落地，力争引进市外资金500亿元，实际利用外资2亿美元，努力在超20亿元重大项目落地上取得突破。加快建立重大项目储备库，深化前期研究，争取金塘澳洲肉牛进境加工等项目提前开工，切实加强项目审批、政策处理和要素保障，形成滚动开发的良好局面。加强对现有企业的全方位优质服务，大力营造亲商、安商、富商的良好投资环境。

强化功能平台建设。海洋产业集聚区、新城、普陀山朱家尖、金塘和六横等五大功能区要发挥主阵地作用，经济增速必须快于全市，更好地支撑新区发展。加快建设海洋科学城、舟山港综合保税区、中国（浙江）大宗商品交易中心。支持县区功能平台建设，强化产业导向和发展特色，统筹项目规划布局。促进定海工业园区、普陀经济技术开发区、岱山经济技术开发区和省级船舶修造产业基地提升发展。推进定海国际粮油园区和普陀浙台经贸合作区、现代商贸流通改革发展试验区、中国舟山国际水产城建设。打造嵊泗国家级列岛风景名胜区和定海国际旅游度假区、普陀国际旅游度假区。加大衢山岛、洋山岛开发力度。

（三）推动经济提质增效。把发展实体经济放到更加突出的位置，推进供给端和需求端协同发力，全力稳增长促转型。

优化提升产业结构。扶持船舶和海工龙头企业，加快产业结构性重组，突破船配高端零部件产业发展，发展船用物料专供服务业，打造船配产品采购交易中心。大力推进“四换三名”和质量强市，实施以“机器换人”为核心的重点技改项目100项以上，加快提升机械、螺杆、汽配等传统制造业。打造智慧海洋应用示范区，推进海洋大数据全产业链发展。加快建设航空产业园。调整房地产结构，取消过时的限制性措施，着力化解房地产库存，促进房地产业健康发展。培育发展涉海建筑业。办好2016国际海岛旅游大会，打造精品旅游示范岛，大力发展邮轮经济，培育海岛民宿、渔家风情、禅修体验、乡村旅游等消费热点。发展健康服务产业，规划建设健康养生城。促进渔农业转型，发展绿色生态精致农业，完成岑港省级现代生态循环农业示范区创建。建设国家远洋渔业基地，推动与葡语系国家渔业合作，建设水产品精深加工集聚区，培育冷链物流等配套服务业。搭建电商公共服务平台，提升舟山产品的网售规模和知名度。抓好定海远洋渔业小镇、朱家尖禅意小镇、沈家门渔港小镇等省级特色小镇建设，培育一批市级特色小镇。

精准服务企业发展。引导全社会形成尊重、关心企业和企业家的良好氛围，制定实施企业减负三年行动计划，建立涉企收费目录清单，清理各种不合理收费，降低企业生产经营成本。加强政银企合作，做强产业基金，鼓励企业对接多层次资本市场，扩大直接融资，加强对正常经营企业的融资服务保障。实施小微企业三年成长计划，大力推进个转企、小升规、规改股、股上市。建立经济信息预警机制，有效防范和化解资金链、担保链风险。对“僵尸企业”通过兼并重组、债务重组乃至破产清算实现市场出清，严厉打击各类恶意逃废债行为。

大力推动创新创业。开展高新科技企业和科技型中小企业“双倍增”行动，支持技术创新、产品创新、管理创新和商业模式创新，出台鼓励企业加大研发投入的政策措施。发挥好高校、公共研发平台等作用，建设新区国家大学科技园。优化升级“5313行动计划”“科创企业助飞计划”，打造山海云间-智库创客总部、科学城创客码头、普陀湾众创码头等众创空间，完善众创服务体系。实施高校毕业生创业引领计划，做大做强市级大学生和青年创业园，建设创业孵化基地。促进创业创新与资本市场的有效对接，吸引市内外人才到舟山创业发展。

建设军民融合创新示范区。规划建设军民融合特色产业园，突出“军转民、民参军”，鼓励企业承接军用产品和参与军品配套科研生产。加快海防基础设施、军地应急应战和动员保障体系建设。全力支持驻舟部队和武警部队改革与建设，做好国防建设、国防教育、国防动员、人民防空和双拥优抚安置等工作，促进军民融合发展。

（四）加快推进体制改革。加大重点领域和关键环节改革力度，实施一批具有牵引作用的改革举措。

创新新区管理体制。探索新区管委会实体运作机制，加快县（区）、功能区管理体制和运行机制改革。强化县（区）、功能区核心制和项目中心制。进一步整合部门职能，建立大部门协调制度。完善对县（区）、功能区、部门和项目指挥部的绩效考核办法。深化海洋综合行政执法，探索创新重点旅游区域综合行政执法体制，加强旅游市场秩序管理。

深化审批制度改革。推进审批流程简化与再造，实行中介服务时间与部门承诺时限捆绑，促进综合验收提速增效，实现以投资项目为重点的行政审批体制改革重大突破，打造全省行政审批环节最少、速度最快、效率最高的城市。推进企业投资项目和便民服务网上申报、网上办理，实现政务服务四级联动。建立政府职能向社会组织转移配套制度，推进中介服务市场化改革。建立权责清单动态调整机制，推进部门职责管理精细化。

推进市场化改革。加快县域经济体制综合改革，推进资源要素配置市场化。深入推进PPP投融资，充分发挥社会资本在基础设施和公用事业建设中的作用。加大政府购买公共服务力度，鼓励社会力量办学、办医、办文化、办养老机构。深化土地海域市场化配置改革，完善陆海联合招拍挂出让模式。整合国企资源，做强海投等四大国有投资公司，探索建立以管资本为主的国有资产监督管理模式。完成公务用车改革。

（五）积极统筹城乡发展。推进城乡建设转型与协调发展，加快建设海上花园城。

加强城市规划建设管理。落实中央城市工作会议精神，推进多规合一，加快城市规划管理创新，加强城市设计和详规控制，推进市体育场、新城隧道复线等建设，完善市政基础配套。提升城市功能，加强楼宇招商，加快集聚城市人气。有序推进定海、普陀城区核心区块有机更新，加快岱山、嵊泗县城建设，推进城中村综合改造。抓好“三改一拆”“四边三化”“交通治堵”，深化“无违建县（区）”创建工作，确保完成省定“无违建”创建任务。加快地下管线信息系统开发建设，加强地下空间规划、开发和利用。创建国家园林城市和森林城市，加大公园和绿地建设，建成绿道网18公里。推进智慧城市建设，提升城市管理服务精细化、人性化水平。创建全国文明城市，提升城乡文明程度。

提升渔农村建设水平。推进六横、金塘小城市和省级中心镇建设。打造美丽海岛升级版，提升村庄规划设计和农房设计质量，推进撤村建居和新型集聚小区建设。抓好新建社区国家级美丽宜居示范村试点工作，保护利用好定海马岙、嵊泗花鸟2个省级历史文化重点村落，打造美丽乡村景观线4条、美丽村口20个。深化“三权”改革，全面推进农村土地承包经营权确权登记颁证工作，完善渔农民合作经济组织体系，创新宅基地使用管理办法。大力发展村级集体经济，完成33个经济薄弱村脱贫转化。完善渔农村社区管理体制。

强化环境生态保护。创建国家生态文明建设示范区，开展“811”美丽舟山建设行动，落实网格化环境监管机制。推进中街山列岛、马鞍列岛特别保护区和嵊泗国家级海洋公园建设。深化舟山渔场修复振兴暨“一打三整治”行动。重视海岛山林迹地改造。推进闲置土地、闲置围填海专项整治，着力解决批而未供、供而未建等遗留问题，垦造耕地6000亩以上。深化“五水共治”，实施消除劣Ⅴ类水整治工程，全面完成“黑臭河”达标治理；建成运行定海污水处理厂三期、小干污水处理厂二期等工程，加强配套管网建设和城区污水管网检测及修复。推进生活垃圾分类收集与资源化处理。强化大气和土壤污染防治。开展排污许可证“一证式”改革试点。

（六）发展民生社会事业。始终把群众的所思所盼作为政府工作的出发点和落脚点。

完善就业和社会保障制度。实施劳动者技能素质提升工程，免费培训技能工人6000名以上，新增城镇就业1万人。健全城乡一体的就业援助制度，动态消除城乡“零就业家庭”。全面完成机关事业单位养老保险制度改革，健全职工和城乡居民大病医疗保险制度，完善城乡一体的全民社保体系。加强低收入群体增收帮扶和精准扶贫。稳步提高低保标准，确保全市低保对象户籍占比达到1.5%以上，低保边缘对象实现应保尽保。支持发展慈善事业。促进残疾人按比例就业，推进残疾人共享小康工程。发展智慧养老，实现居家养老服务有形覆盖和有效覆盖。

推进公共服务标准化均等化。加快义务教育优质均衡发展，统筹布局教育资源，基本完成教育基本现代化县（区）创建。推进学前教育就近入园。支持浙江大学海洋学院、浙江海洋学院发展，办好新区旅游与健康职业学院，完善浙江国际海运学院办学设施。完成舟山航海学校迁建改造工程，基本建成舟山绿城国际学校。启动海洋健康城市建设。深化“双下沉、两提升”工作，提高基层医疗服务能力。社区卫生服务中心签约服务开展率达到100%新建渔农村文化礼堂25家以上，加强基层综合文化服务中心建设。加快舟山传媒创意中心建设进度。做大“淘文化”平台，筹建“数字文化发展中心”。筹办第三届省体育大会。建成运行新区公共服务与监管中心。

全面推进平安舟山建设。围绕“平安金鼎”目标，深化“铁桶固防”，健全海岛立体化社会治安防控体系，严厉打击各类突出违法犯罪，推进反恐工作常态化，探索建设“警务云”大数据平台，全面完成杭州G20峰会“护城河”防护任务。提升城乡社区管理水平，打造社区睦邻中心，完成村级换届选举试点。深化“网格化管理、组团式服务”，加强重大决策社会风险评估。全面开展“七五”普法，构建公共法律服务体系。健全多元化纠纷解决机制，完善社会管理信息系统平台。全面推进户籍制度改革。加快“三小一市场”和进口食品市场整治提升，深入开展餐桌安全治理行动三年计划，推进企业诚信体系和社会信用建设。加快北斗船载终端和自动识别系统更新工作，建成海洋灾害应急指挥平台。加强三防工作，全面排查地质灾害隐患，抓好渔业、船舶、大桥、水陆交通、油品等重点领域的安全生产，完善突发公共事件预警信息发布体系。

各位代表，经过广泛征求意见，今年我们继续确定了十方面民生实事。市政府将进一步健全工作机制，切实把民生实事办好。

1.抓好“菜篮子”工程。建成蔬菜基地2000亩，提升改造1000亩；创建6家省级放心农贸市场；新建菜篮子直供店10个。

2.加强社会保障帮扶。开展渔民养老保险工作；80%低收入渔农户家庭人均年纯收入达到1万元；新增养老机构床位600张，新建居家养老照料中心50家；建成10个红十字博爱家园。

3.加强水环境治理。完成10条25.8公里的黑臭河治理；完成12座县级以上饮用水源一级保护区隔离工程建设；基本建成虹桥水厂深度水处理工程。

4.加大医疗惠民力度。建立5个远程医疗服务中心，服务网络覆盖所有乡镇卫生院和社区卫生服务中心；开展渔农民流动医院免费巡回医疗服务100次以上；启动舟山医院感染病房建设。

5.加强污染治理。淘汰改造燃煤小锅炉220台；对首批全市30%重点危废单位实行动态跟踪管理；实现市控以上排污单位污染物排放、行政处罚及环境信用等信息公开。

6.推进安居工程。改造城区D级危房53幢1018户，交付入住公共租赁房和经济适用房2459套。

7.提升渔农村环境。10个社区（村）建成美丽海岛精品社区，15个社区（村）开展生活垃圾分类收集和处理。

8.方便群众出行。建成港湾式停靠站150个，新增或更新公交车36辆，新增客船4艘，建成陆岛码头6座，推出公交出行服务手机APP。

9.丰富群众文体生活。建成12个基层体育休闲公园，开展“百团百艺进文化礼堂”惠民演出活动250场。

10.促进教育公平。义务教育学校标准化率达到90%，普惠性幼儿园覆盖面达到85%以上，70%以上学校达到“美丽学校”标准。

各位代表，非常之时，必须拿出非常状态，凝聚非常之力。我们要按照市委“树标杆、补短板、求突破、走前列”的要求，始终把为企业服务、为民办实事作为政府履职的重中之重，作为衡量各级政府是否敢于担当的重要标准，努力在打造全省“审批环节最少、速度最快、效率最高”城市上有突破，建设服务政府；始终践行敢为人先的创业精神，先行先试，大胆突破，坚决摒弃传统路径依赖，闯出新区发展新天地，建设创新政府；始终坚持雷厉风行抓落实，敢闯敢干，敢抓敢管，坚决整肃庸政懒政怠政，破除“中梗阻”，加大简政放权力度，建设高效政府；始终以法治思维和法治方式推进政府工作，自觉接受人大监督、政协民主监督、司法监督和各方面监督，建设法治政府；始终严守政治纪律和政治规矩，坚决整肃“四风”，坚决惩处各类腐败行为，厉行节约反对浪费，建设廉洁政府。

各位代表，干在实处永无止境，走在前列要谋新篇。让我们更加紧密地团结在以习近平同志为总书记的党中央周围，在省委、省政府和市委的坚强领导下，围绕本次大会确定的目标任务，齐心协力，锐意进取，克难攻坚，为实现发展新跨越、建设魅力新舟山而努力奋斗！
